# Interactions between CNS regulation and serotonergic modulation of crayfish hindgut motility

**DOI:** 10.1098/rsos.250094

**Published:** 2025-06-18

**Authors:** Spandan Pathak, Norma Peña-Flores, Phillip Alvarez, Jenna Feeley, Reza Ghodssi, Wolfgang Losert, Jens Herberholz

**Affiliations:** ^1^ Institute for Physical Science and Technology, University of Maryland at College Park, College Park, MD, USA; ^2^ Neuroscience and Cognitive Science Program, University of Maryland at College Park, College Park, MD, USA; ^3^ Department of Electrical and Computer Engineering, University of Maryland at College Park, College Park, MD, USA; ^4^ Institute for Systems Research, University of Maryland at College Park, College Park, MD, USA; ^5^ Department of Physics, University of Maryland at College Park, College Park, MD, USA; ^6^ Department of Psychology, University of Maryland at College Park, College Park, MD, USA

**Keywords:** optical flow, intestinal motility, invertebrate, serotonin, gut–brain axis

## Abstract

Motility is a critical function of the gastrointestinal (GI) system governed by neurogenic and myogenic processes. Due to its major role in maintaining homeostasis, overlapping mechanisms have evolved for its adaptive operation including modulation by the central nervous system (CNS), enteric nervous system (ENS) and intrinsic pacemaker cells. Our understanding of the modulatory mechanisms that underlie intestinal motility remains incomplete. Crayfish provide a tractable *ex vivo* model to study the interplay between CNS and neurochemical regulation of GI motor patterns. Our study investigated the effects of CNS denervation and exogenously applied serotonin (5-HT) on crayfish hindgut motility. Multiscale spatial measurements showed stable motility parameters throughout 90 min of control conditions. Denervation, i.e. separating the gut from the CNS, resulted in a significant decrease in the magnitude and synchrony of hindgut contractions, while preserving the underlying frequency and directional bias of the waves. Subsequent application of 5-HT to the denervated preparation enhanced motility but disrupted spatiotemporal coordination. Treatment with TTX (a sodium channel blocker) had minor impacts on motility metrics, indicating a prominent role of myogenic mechanisms. Our model provides a multiscale analysis framework to dissect CNS and interrelated neurochemistry contributions to GI motor dynamics.

## 1. Introduction

The nervous system is an information-processing system involved in sensing, integrating and controlling all bodily functions. From interpreting internal and external sensory information received through afferent neurons to coordinating behavioural responses through muscular activation, the central nervous system (CNS) plays an integral role as the body’s command centre. While most bodily functions are controlled by the CNS, many aspects of ‘day-to-day’ operation of an organism are only weakly supervised. They are instead regulated by *local* neural populations and associated cellular systems that receive sensory input and coordinate motor and neurosecretory output, especially in the gastrointestinal (GI) tract and heart [1].

Here, we investigate GI motility which allows intestinal contents to mix and propel along the GI tract and is a critical function of the GI system to maintain homeostasis. Uniquely, the GI system is the only organ system to have evolved an independent nervous system—the enteric nervous system (ENS)—which in concert with the CNS and local biochemical environment regulates its sensory, motor and secretory functions [2]. In mammals, this complex phenomenon is modulated by overlapping and cooperating neural systems, namely the intrinsic ENS, extrinsic innervation by the CNS and pacemaker-like cells known as interstitial cells of Cajal (ICCs) [3]. Disorders that affect GI motility such as irritable bowel syndrome (IBS) have a neuropathic component where dysfunctional CNS and ENS neurons lead to intestinal dysmotility [4,5]. Compromised gut motility prevents proper nutrient absorption and can be potentially fatal [6]. Notably, functional disorders of gut–brain interaction are prevalent among more than 40% of the global population [7]. Despite the pressing need to better understand the mechanisms underlying GI dysmotility in various diseases [8], the development of new treatments has been limited [9].

Conveniently, basic functions of the GI system such as intestinal motility are highly conserved across evolution. The general structure and neurochemistry of the GI tract is found across species as diverse as hydra, arthropods, octopuses and humans [10–12]. Thus, simpler animal models with more tractable and accessible GI systems provide a promising alternative to study the mechanisms regulating gut motion.

In particular, decapod crustaceans have a simple, accessible and well-characterized anatomy that allows dynamic study of the interplay between the GI tract and the CNS. The posterior segment of their digestive system, the hindgut, is located in their abdomen and has a reduced anatomy and physiology compared to vertebrates. The hindgut connects to the abdominal nerve cord (ANC), which is part of the CNS, via a single nerve (‘nerve 7’) in crayfish [13] or a paired nerve (‘paired intestinal nerve’) in lobsters [14]. The crayfish hindgut is innervated by two nerve plexuses, an outer plexus originating from the terminal abdominal ganglion and an inner plexus that runs in between the circular and longitudinal muscle layers. While the outer plexus arises from the nerves projecting from the terminal abdominal ganglion, whether the inner plexus has an extrinsic or intrinsic origin is yet unknown, even though groundwork on these animal models was laid almost a century ago [15]. Innervation from the ANC may be ethologically useful as it contains well-characterized neuronal circuits that control important behaviours like escape and swimming [16].

Moreover, crayfish, a group of inexpensive, wildly available freshwater crustaceans, are particularly well suited for *ex vivo* imaging of hindgut motility. Like the mammalian GI tract, even without external stimulation or CNS innervation, the crayfish hindgut spontaneously manifests wave-like movement caused by the contraction of circular and longitudinal muscles [17]. These rhythmic contractions are produced by putative myogenic pacemakers [18], and hypothesized to be modulated by the CNS [14] via several neurotransmitters including serotonin [19], dopamine [20], glutamate [21] and neuropeptides [22,23]. The crayfish preparation is particularly robust as the hindgut and connected ANC can remain active for several hours when pinned down in a dish filled with crayfish saline, allowing *ex vivo* real-time study of hindgut motility through direct imaging.

Serotonergic modulation of hindgut motility is well documented; however, the historical view on the underlying mechanism has been challenged. In mammals, 5-HT is produced endogenously by enterochromaffin cells in the intestinal wall [24], which locally release most of the body’s 5-HT as well as exogenously by extrinsic neural processes [25]. This combination of endogenous and exogenous 5-HT has complex effects on a vast array of differentially localized 5-HT receptors including 5-HT1, 5-HT3, 5-HT4 and 5-HT7 [26,27]. The notion that endogenously released 5-HT acts on enteric neurons to regulate GI motility has been challenged by several experiments. For example, overall intestinal motility and gut transit are only weakly affected after enteric 5-HT production and/or mucosal release are ablated, questioning the importance of gut-derived 5-HT in controlling gut motility [28,29]. Moreover, certain antagonists selective for 5-HT receptors (e.g. 5-HT_3_ and 5-HT_4_) block gut motility even in the absence of endogenous 5-HT [30,31]. Since 5-HT receptor subtypes are expressed in the gut epithelium, it has been suggested that exogenous 5-HT affects gut motility directly via these receptors, depending more on neuronal 5-HT than EC-released 5-HT [32,33].

In crayfish, exogenous serotonergic modulation of hindgut motility and serotonergic innervation of the hindgut have been partially characterized [19,34]. Particularly, 5-HT receptors 5-HT1α and 5-HT2β have been shown to be differentially expressed along the crayfish hindgut [34]. Moreover, bath application of 5-HT —mimicking neurohormonal activity—has been shown to modulate hindgut motor activity, increasing amplitude and frequency, in an isolated crayfish hindgut [19,34]. Thus, 5-HT receptors may have spatially differentiated and concentration-dependent effects on crayfish hindgut motility, as observed in mammals. Despite the potential for understanding gut motor dynamics using a crayfish model, the invasive and low-resolution nature of traditionally used methods (e.g. force transducers) have rendered dynamic changes in the physical properties of the hindgut and their corresponding motility patterns largely unknown.

In this study, we used neuropharmacology combined with optical flow analysis—a non-invasive machine vision technique that extracts pixel level (μm) spatiotemporal (ST) flow fields—to investigate the effects of hindgut denervation and subsequent 5-HT application on hindgut motility. We predicted both CNS and 5-HT would impact different aspects of hindgut motility, revealing CNS modulation of hindgut motor control as well as parallel mechanisms intrinsic to the hindgut and independent of CNS control. To extract multiscale motility parameters related to hindgut motion, we applied Fourier analysis to our ST flow fields to better understand the underlying time scales as well as the overall strength of hindgut motion. Previous work has shown that these dynamics can be combined to track cellular or tissue movement across different spatial and temporal scales [35,36]. By providing a multiscale analysis framework, our study lays the groundwork for the real-time investigation of the cellular-molecular mechanisms governing CNS and serotonergic modulation of gut motility in a crayfish model. In addition, our results will inform studies in other organisms and improve our limited understanding of the complex mechanisms underlying GI motility control.

## 2. Material and methods

### 2.1. Animals

All animals used for the 5-HT experiments were adult male crayfish (*Procambarus clarkii*, *n* = 30) obtained from commercial suppliers (Niles Biological Inc. or Carolina Biological Supply Co.) of similar weight and size and length (17.1 ± 8.2 g, 8.0 ± 1.2 cm). Animals were housed in communal tanks (30 gallons; 76 × 30 × 30 cm, length × width × height) with 3−10 other crayfish and kept on a 12-h light : dark cycle at 22 ± 1.5°C. They were fed two shrimp pellets (Ocean Nutrition Formula One Marine Pellets) twice a week per animal in the communal tanks.

To ensure feeding was uniform and the animals’ hindguts would be empty prior to dissection, crayfish were taken from communal tanks 3−4 days after their last communal feeding and individually isolated in smaller tanks (2 l; 20 × 12 × 13 cm, length × width × height). They were fed one shrimp pellet on the day of isolation and kept isolated for four days without further feeding prior to experiments.

### 2.2. Dissection

**Figure 1.**
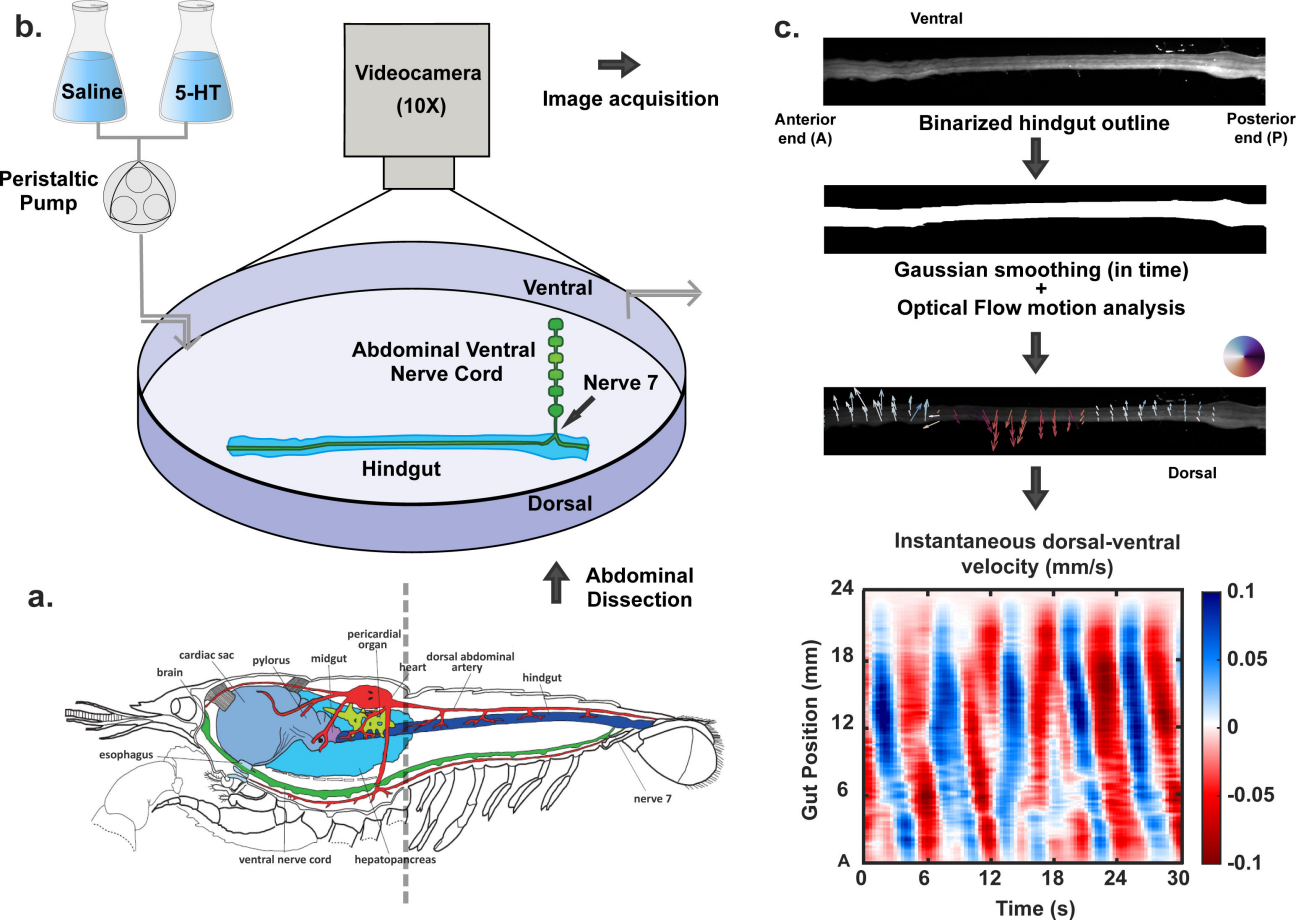
Schematic of experimental setup and image analysis workflow. (a)The hindgut–nerve cord preparation was dissected from the crayfish abdomen (illustration used with permission [19]). For the 5-HT experiments, a set of 30 individual experiments was collected and analysed from 30 individual animals. Each animal was only used once. The average weights and sizes for each condition (*n* = 6 per condition) were: saline on intact preparation 17.9 g ± 10.2 g/ 8.2 cm ± 1.4 cm; saline with N7 neurectomy 19.7 g ± 10.1 g/ 8.2 cm ± 1.6 cm; 1 μM 5-HT 16.7 g ± 6.5 g/ 8.0 cm ± 0.9 cm; 10 μM 5-HT 17.0g ± 5.0 g/ 8.0 cm ± 1.0 cm; 100 μM 5-HT 16.5 g ± 8.4 g/ 7.9 cm ± 1.1 cm. Average animal weights and sizes for the TTX experiments were: 14.7 g ± 1.9 g/ 8.1 cm ± 0.3 cm; (b) The *ex vivo* hindgut–nerve cord preparation was pinned on a sylgard-lined petri dish and video recorded at 10× magnification under three sequential conditions 30 min each) (other than TTX): 1. perfusion of crayfish saline (baseline) on intact preparation; 2. perfusion of crayfish saline on hindgut isolated from CNS by N7 neurectomy; 3. perfusion of crayfish saline (control) or perfusion of 5-HT at 1, 10 or 100 µM. A second control experiment video recorded the intact preparation superfused with saline throughout 90 min. (c) Pixel intensity-based thresholding was used to binarize the movies, extracting the hindgut outline in the process. Optical flow analysis was performed on the temporally smoothed time-lapses to capture movement of the hindgut. Arrow lengths quantify the magnitude of motion at different spatial locations while different directions are represented by colour-coded arrows on a circular map. Finally, all dorsal–ventral (DV) movements (perpendicular to the central axis of the gut) were averaged along the DV axis to obtain mean instantaneous velocities. Their changes in time were captured from the resultant ST plots with blue and red bands indicating periodic DV movement along two directions. Further, motility parameters like wave speed, spatial span and directionality were extracted from the bands. Detailed description of the image analysis pipeline can be found in §2.

Animals were anaesthetized on ice for 15−25 min before their abdomens were removed. To expose the nerve cord, the ventral carapace was first cut bilaterally to sever the lateral nerves projecting from the nerve cord and lifted away with forceps stopping at the anus. To expose the hindgut, the dorsal carapace was also cut bilaterally down to the tail fan and removed down to the most posterior abdominal segment. The dorsal artery running along the hindgut tract was then exposed and pulled away from the hindgut. To isolate the terminal portion of the nerve cord while preserving its connection to the hindgut, the ventral carapace was lifted, and the nerve cord was pulled away from it, carefully cutting the lateral nerves projecting from the terminal ganglion except for nerves 5, 6 and 7 that project to the telson, anal, and hindgut musculature, respectively. The anus was then detached from the tail cuticle and the surrounding abdominal musculature was removed. The hindgut attached to the nerve cord by N7 was taken out and placed in crayfish saline [37]. At this stage, all remaining muscle, arteries as well as nerves 5 and 6 were removed. All other nerves projecting from the terminal ganglion except for N7 were trimmed as close to the ganglion as possible. Finally, the hindgut–nerve cord preparation was pinned out in a sylgard-lined petri dish with new crayfish saline. Insect pins were used to anchor the posterior and anterior ends of the hindgut, as well as the anterior ganglion of the nerve cord, while finer pins were used to tether the lateral nerves projecting from each abdominal ganglion of the nerve cord (figure 1).

### 2.3. Physiology

Video recordings of the preparation were taken at 30 frames per second for 90 min using a monochrome CMOS camera attached to a dissecting microscope (10× magnification). Control experiments continuously superfused the intact preparation with crayfish saline at a rate of 5 ml min^−1^ (Thermo Scientific, FH100 Peristaltic Pump) to assess viability during the total duration of the experiment. Superfusion rate was recalibrated weekly. All other experiments (other than TTX) included three sequential conditions (30 min each): (i) intact preparation, i.e. the hindgut and ventral nerve cord connected by the intestinal nerve (N7), superfused with crayfish saline (baseline); (ii) hindgut isolated from the CNS by severing N7 with microscissors while perfusing with crayfish saline; and (iii) isolated hindgut superfused with crayfish saline (control) or 3 different 5-HT concentrations (1, 10 or 100 μM).

To determine the separate contribution of neurogenic versus myogenic components to gut motility, electrophysiology experiments using 10^−7^ M TTX (tetrodotoxin, a sodium channel blocker) diluted in crayfish saline were performed on a denervated hindgut (*n* = 1) and on intact hindgut–nerve cord preparations (*n* = 5) while stimulating the anterior nerve cord connectives with silver-wire electrodes or with no electrical stimulation. Experiments included the following sequential conditions: (i) superfusion with saline (30 min baseline); (ii) superfusion with TTX or saline (30 min); and (iii) washout with crayfish saline (1 or 3 h).

### 2.4. Spike counting

Action potentials (spikes) evoked in the ANC and N7 by placing silver-wire electrodes on the anterior ANC were recorded by a second set of electrodes placed on the posterior ANC or by a suction electrode on N7. Spontaneous activity was recorded with the same electrode arrangement but without any stimulation. Clampfit software (Molecular Devices) was used for spike counting in TTX experiments and controls. The event detection tool was selected, and a threshold search was performed. For experiments with episodic sweep settings, thresholds (positive or negative) were set manually above baseline noise (±0.5 mV or higher). After level markers for re-arming and trigger were set, a detection window of 30 ms was selected with the placement of cursors at defined time points. The first cursor was set on the baseline level immediately following the stimulus artefact, and the second cursor 30 ms after the first cursor. Threshold rejection was used sparsely and only if large artefacts were present in the recorded traces.

To match our video analysis, the last 15 min of each phase (saline, TTX, washout) was analysed. The number of events (i.e. spikes) detected by the program for each phase was displayed in the event viewer and recorded in the results. For episodic recordings without stimulation, a 200-ms window was used to count spontaneous spikes. When using gap-free recording mode without electrical stimulation (control), spontaneously occurring spikes were counted during time intervals that matched the episodic and video recordings of the saline and TTX phases as well as a later time point (i.e. 15−30, 45−60 and 75−90 min). The same procedure was applied for spike counting in both the abdominal nerve cord (ANC) and N7 recordings.

### 2.5. Image analysis workflow

#### 2.5.1. Post-processing of images

The raw time-lapses were cropped using ImageJ to zoom in on the entire hindgut region. Transition frames accounting for denervation and 5-HT application were removed. The mean time elapsed for these cropped transition phases was 29 s (denervation) and 8 s (5-HT), respectively. We found hindgut movements happened at relatively long timescales, and hence for our analysis purposes, reduced the frames per second (f.p.s.) of raw images from 30 to 3 by skipping every nine frames. All down-sampled movies were smoothed in time using the Simoncelli (five consecutive frames with the weights: 0.036, 0.249, 0.431, 0.249 and 0.036) smoothing filter [38]. Results were not sensitive to the exact smoothing parameters given that five-frame smoothing only affects the power spectrum at higher frequencies, and our characteristic motion occurred at much lower frequencies. Spatial smoothing was avoided to retain sharp features for optical flow analysis.

#### 2.5.2. Identifying hindgut outline and ST flow fields using optical flow

The hindgut outline for each timeframe was extracted from raw cropped images using ‘*strel’* and ‘*imerode’* in MATLAB. Median pixel intensity of every image was used for the binarization threshold. Optical flow analysis was performed on the smoothed and cropped time-lapses by the Lucas–Kanade method [39]. The optical flow weight matrix around each image location was a Gaussian with a s.d. of 2 pixels. A reliability threshold of 0.01 was also imposed to ensure movements only within the gut region were considered. Sub-threshold flow vectors were made zero. We confirmed that the size and shape of the Gaussian filter did not significantly alter motion capture and downstream analysis. Subsequently, all lateral optical flow vectors within the extracted hindgut region were averaged along the dorsal–ventral axis of that frame. As a result, we obtained dorsal–ventral motility plots (figure 1), i.e. averaged lateral motion along the length of the hindgut as a function of time. For visualization, we used Gaussian smoothing on the raw flow vectors to obtain movement directions and magnitude at a larger spatial scale and ignore spurious localized effects due to random fluctuations in pixel intensity. A threshold was also applied to the resultant vector magnitudes to avoid background noise.

#### 2.5.3. Power analysis of flow fields

Applying fast Fourier transform (FFT) on the dorsal–ventral motility plots against the time axis, we obtained two-dimensional power spectral density (PSD) of lateral hindgut motion. Averaging along the spatial axis, we derived the squared amplitude corresponding to all frequency components below the Nyquist frequency of 1.5 Hz (sampling frequency is 3 Hz). In signal processing terms, power is the sum of the squared amplitudes of all time-domain or frequency-domain samples divided by the signal length. We could identify characteristic frequency peaks (corresponding to the highest power value) and harmonics for different phases of each experiment. Similarly, using FFT against the spatial axis of motility plots led to instantaneous power. Here, the sum of the squared amplitude of all frequency components has been interchangeably used with the term ‘power’ as higher amplitudes of the input signal (i.e. the ST flow field) correspond to higher mechanical power of movement.

#### 2.5.4. Frequency peak of PSD

The overall power embedded in a signal has contributions from a regular, periodic component (rhythmic power) and an irregular, noise-like component of the signal. Relative rhythmic power (rhythmic power divided by total power) from power spectral density quantifies the contribution of the peak frequency band to the overall power. It is regarded as a measure of temporal coordination in the input signal. For pure white noise, the PSD should be a flat horizontal line whereas, for a signal with a purely single frequency, the PSD would be a Dirac-delta function, and thus the relative rhythmic power near the peak should be 1. Based on this, we quantified the coordination of hindgut movement with the metric, relative rhythmic power. To define a frequency band near the peak, we used a window of 0.04 Hz symmetrically on both sides. We noted that the resulting analysis does not change qualitatively based on the chosen peak width within a reasonable range.

#### 2.5.5. Lateral hindgut wave identification

From DV flow fields, we applied area, length and speed thresholds to extract individual lateral waves. First, we applied a Gaussian filter with a s.d. of 2 pixels to extract regions having instantaneous speed above a chosen threshold (top 30% speed value, pooled across all movies). Then we used ‘*bwareaopen*’ and ‘*bwlabeln*’ (from MATLAB) to find and label all connected components (above the area threshold of 100 pixels) within our identified region. Finally, we extracted individual regions having a spatial span of more than 1/5th of hindgut length, separating large-scale (spatial and magnitude) movements from small, local fluctuations in the process. Upon identification, we used ‘*bwskel*’ (from MATLAB) to extract the central axis of the wave regions. The spatial span divided upon the temporal span (i.e. slope of the extracted bands) provided us with the lateral speed of the wave. A wave without any ‘temporal span’ indicated parts of the hindgut moving in synchrony without any waveform passing through. We used the term ‘mixed wave’ for referring to these motility patterns. We also identified the directionality of these lateral waves by labelling all anterior–posterior (AP) movements with positive wave speed and posterior–anterior (PA) movements with a negative wave speed, respectively.

#### 2.5.6. Choosing time-windows for statistical analysis

In an experimental run, we only considered the last 15 min of the first two phases and the first 15 min of the third phase for our analysis (15−30, 45−60 and 60−75 min from the start, respectively), as we aimed to capture the response of the hindgut upon serotonergic excitation. We observed that any increase in power is short-term and thus, for quantification, the hindgut motility was analysed immediately before and after the application of 5-HT. We also ran statistical tests to find differences between 15-min intervals among the treatments, which supported our rationale for choosing such discrete time intervals (see electronic supplementary material, figure S1). For the TTX experiments, we again analysed the final 15 min of the first two phases (Baseline saline: 15−30 min, TTX: 45−60 min from the start). However, for the crayfish saline wash phase, we used the final 15 min of the phase (105−120 min), as TTX is known to require a longer duration to be fully washed out.

#### 2.5.7. Statistical testing for inter- and intra-treatment effects on hindgut motility parameters

Shapiro-Wilks normality tests were performed to test distribution of motility parameters corresponding to each kind of treatment (*n* = 6). Thereafter Friedman and subsequently Wilcoxon’s (on those passing Friedman with *p* < 0.05) signed-rank tests were performed for pairwise comparisons between different phases (baseline versus second phase, baseline versus third phase, second phase versus third phase), for each unique treatment (see electronic supplementary material, tables S1 and S2). All saline + N7 cut movies (*n* = 24) were pooled together to test the effects of N7 cut concerning the baseline. Similarly, all saline + N7 cut + 5 HT movies (*n* = 18) were pooled together to test the effects of 5-HT application concerning the baseline as well as the N7 cut phase. For TTX experiments, the number of repeats was too low for feasible statistical analysis among multiple treatment groups. *p*-values are reported in figures for 0.1 > *p* > 0.05, * for 0.01 < *p* ≤ 0.05, ** for 0.001 < *p* ≤ 0.01, *** for 0 < *p* ≤ 0.001.

## 3. Results

### 3.1. Image analysis workflow captures complex dynamics of hindgut movement in crayfish and quantifies motility parameters

**Figure 2.**
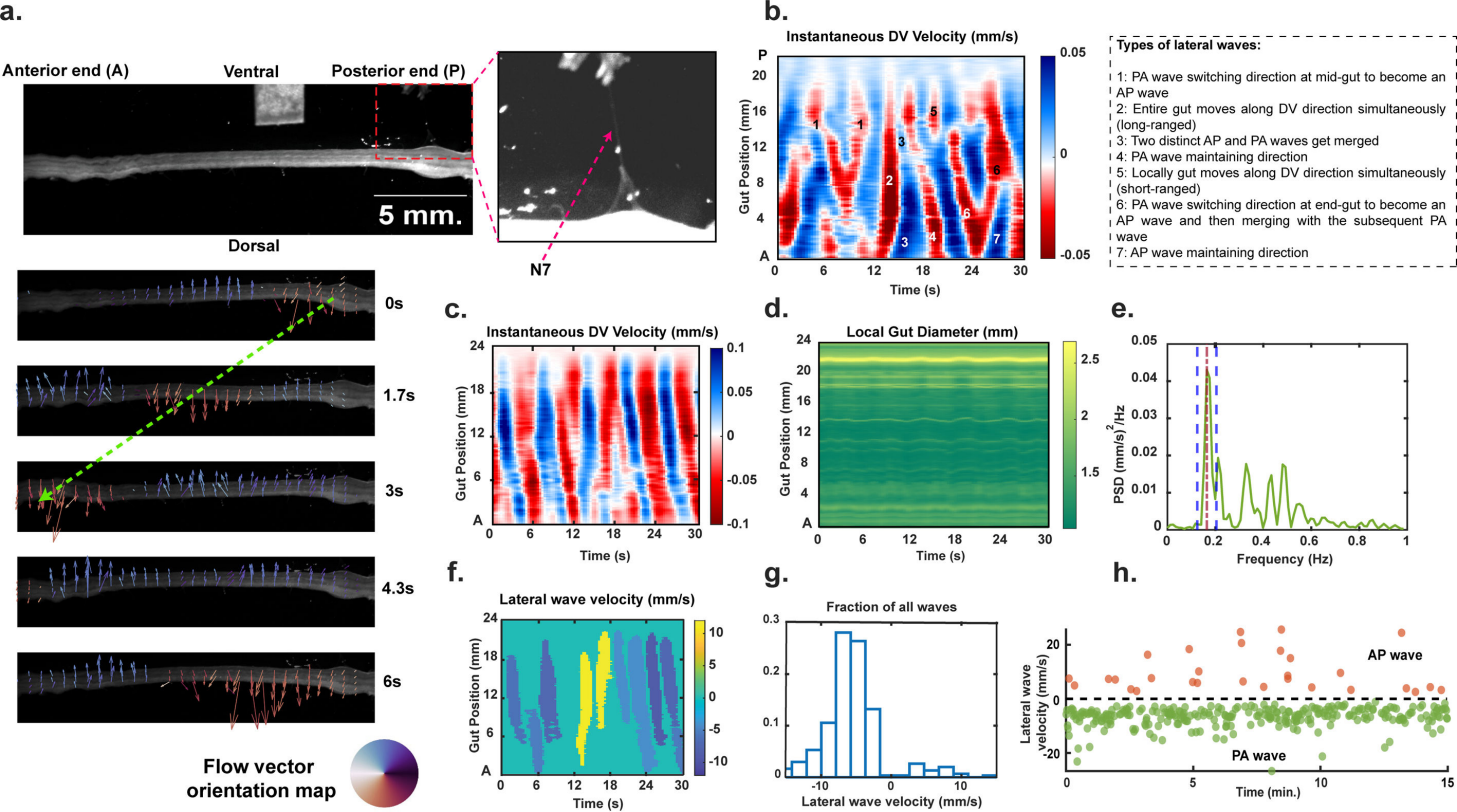
Optical flow calculations capture the dynamics of hindgut movement. (a) Demonstration of motion capture workflow from isolated crayfish hindgut movies. Subsequently, a series of timeframes overlaid with optical flow vectors indicate wave-like propagation of local DV movements. (b) DV motility plot depicting different kinds of lateral waves. (c) Representative DV motility plot (saline treatment) for 30 s of gut movement under saline treatment. (Lower gut position values correspond to the anterior end.) The colour axis represents the velocities, with positive and negative values indicating movements towards the ventral and dorsal direction, respectively. (d) Local gut diameter as a function of time for the same gut movie as (c). (e) Power spectral density of the motility plot from (c) averaged along the spatial axis. (f) Identification of lateral gut waves based on instantaneous DV speed and spatial span threshold. Wave speed is calculated using the slope of the extracted wavebands. The colour axis represents the wave velocities with positive values indicating movements from the anterior to the posterior direction (i.e. AP waves). (g) Probability distribution of lateral wave velocities from a saline treatment of 15 min (same movie as (c–f)). (h) Lateral wave velocities of individual waves versus time from (c).

To investigate the complex hindgut motion observed in crayfish, we developed an image analysis workflow that captures and quantifies motility parameters from brightfield images (as shown in figure 2). We used ‘optical flow’, a widely used machine vision technique [36], to detect movements at individual pixel level, constructed ST flow fields to obtain averaged movement along the DV direction, and applied Fourier transformation to reduce the complex motion down to a few meaningful motility parameters. The workflow is further described in §2.

Figure 2a shows a representative crayfish hindgut image. The principal or AP crayfish gut axis has the nerve cord N7 attached near the posterior (P) end (pointed at by the red arrow). Thereafter, through a sequence of timeframes, optical flow analysis demonstrates wavelike motion, at different regions of the gut. The length and direction of the arrows quantify the magnitude and direction of instantaneous motion at each spatial location.

Most movements occurred in the DV direction and very few flow vectors were aligned with the AP axis indicating a much prominent role of longitudinal muscle contractions. In successive timeframes, we observed a wavelike progression of localized movements along the AP axis which is perpendicular to the direction of instantaneous velocities. In this representative case, we noted a localized downward waveform traveling from the posterior to the anterior end (shown in form of the dashed green line) in a period of 3 s. We denoted these lateral wave-like movements along the AP axis as ‘lateral waves’. Figure 2b demonstrates a variety of these long- and short-ranged wave patterns. We found waves travelling along both directions (AP and PA), waves without any clear directionality (‘mixed waves’), two successive waves merging, and waves changing directions at the midpoint of the gut. In figure 2c, we constructed an ST motility plot from 30 s of baseline (saline) treatment that captured the wavelike progressions of mean DV movement along the length of the gut. We observed slanted alternate blue and red bands in this chosen time-window, indicating gut movement in a coordinated and periodic manner. Figure 2d shows no major periodic changes in local gut diameter indicating minimal circular contractions. To quantify and characterize this complex motion, we next applied two-dimensional fast Fourier transformation to the ST motility plots and in Fourier space, averaged the contributions from all gut locations. The power spectral density (PSD) from figure 2e demonstrates how different frequencies contributed to the rhythmic nature of the gut movement. The total area under this PSD curve (power) is related to the amplitude of gut movement and is characteristic of the mechanical power of the system undergoing motion. We observed a sharp peak in the resulting PSD close to a characteristic frequency of 0.2 Hz (shown by the brown dashed line). The normalized area around the peak is a measure of the temporal coordination of movement. To extract further information about lateral waves, we measured the speed of the waves from the space–time motility plots. The slope of the extracted bands provides us with the travelling wave speeds (figure 2f), where we can notice different directions of waves travel within the given time window (AP movement is denoted by positive velocity values). Using a longer period of gut motion (15 min of saline treatment on the hindgut preparation), we found a bimodal distribution of velocities (figure 2g) with a clear preference for PA movement. Figure 2h highlights the large number of lateral waves observed in a typical experiment and points towards the infrequent directional switching of these waves.

### 3.2. Hindgut movement decreases upon denervation and increases after 5-HT application

**Figure 3.**
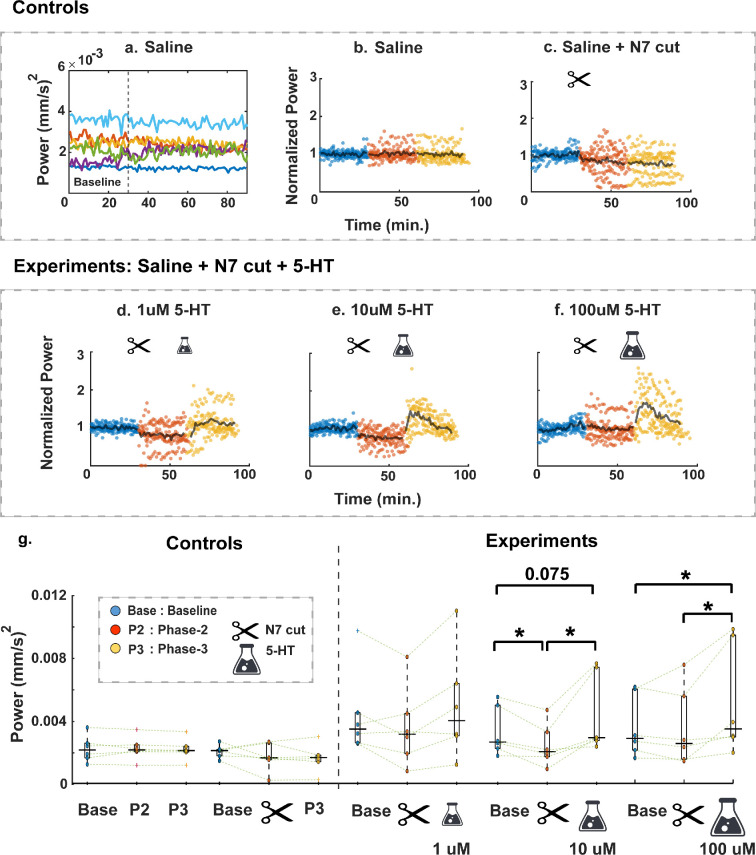
Hindgut movement over experimental runs (for varying treatments and all repeats). (a,b) Combined power and normalized power versus time for the six controls without denervation (the first 30 min are regarded as the baseline of each experiment). (c) Normalized power versus time for the six saline + N7 cut controls. (d–f) Normalized power versus time for all experimental runs. *n* = 6 for each of the saline + N7 cut + 5 HT concentrations. Blue, red and yellow correspond to three different phases of an experiment. Each dot represents characteristic power of lateral motion (averaged over 1 min) normalized by the mean baseline power for that experiment. Black lines show the median normalized power tracks across all experiments with similar treatment. The N7 neurectomy and 5-HT application were performed at 30 and 60 min from the start, respectively. (g) Average power during three different phases of an experiment for individual treatments (*n* = 6). Values for baseline, N7 neurectomy and 5-HT phases taken from the second, second and first 15 min of each phase, respectively. *p*-values were obtained using Friedman and Wilcoxon’s signed rank test criteria. Boxplot edges indicate the 25th−75th percentiles of the sample data and solid black horizontal lines indicate median values.

Next, we studied the characteristic power of gut movement throughout an entire experiment (90 min). We averaged instantaneous power using short time windows of 1 min and obtained a time track for each experimental run. Since the first 30 min was always the saline treatment, we defined it as the baseline for each experiment (figure 3a). During baseline, the hindgut was connected to the nerve cord and superfused with saline. To avoid variance in power due to animal variation, we scaled each time track using the mean baseline power. We denoted the re-scaled dimensionless quantity as ‘normalized power’.

Overlaying six individual time-tracks (*n* = 6 for each treatment; figure 3b–f) with the medians in the centre (given by the black horizontal lines), revealed large-scale temporal variations. In the baseline (saline) control, there were no distinct changes in power levels throughout, suggesting steady dynamics over 90 min (figure 3a,b). In serotonergic modulation experiments (figure 3d), we observed that after denervation in phase 2, when N7 is cut and the hindgut is disconnected from the CNS, power decreased slightly, which was again regained after the application of 5-HT to the isolated hindgut (figure 3d–f). However, this peak in mechanical response was not sustained, and for the two higher 5-HT concentrations (10 and 100 μM), power eventually fell close to baseline levels again. For the lowest concentration of 1 μM, we observed a delayed rise in power after the application of 5-HT. Interestingly, we also noticed that there was much variability in intra-experiment power levels once the nerve cord was cut and this was maintained even after 5-HT was added (indicated by a much wider distribution of the red and yellow dots). In the first phase, this variability in power was minimal. In figure 3g, we show that application of 5-HT drove power past baseline levels in a dose-dependent manner. The experiments with the highest concentration of 5-HT (100 μM) led to power levels that were significantly higher than both the baseline (*p* = 0.046) and N7 cut phase (*p* = 0.028). In the medium concentration (10 μM), power levels were slightly higher than the baseline (but statistically non-significant, *p* = 0.075) and significantly higher than the N7 cut phase (*p =* 0.028)*.* For the lowest concentration of 1 μM 5-HT, such changes were not significant.

### 3.3. Hindgut motility is influenced by both CNS coordination and abundance of 5-HT

**Figure 4.**
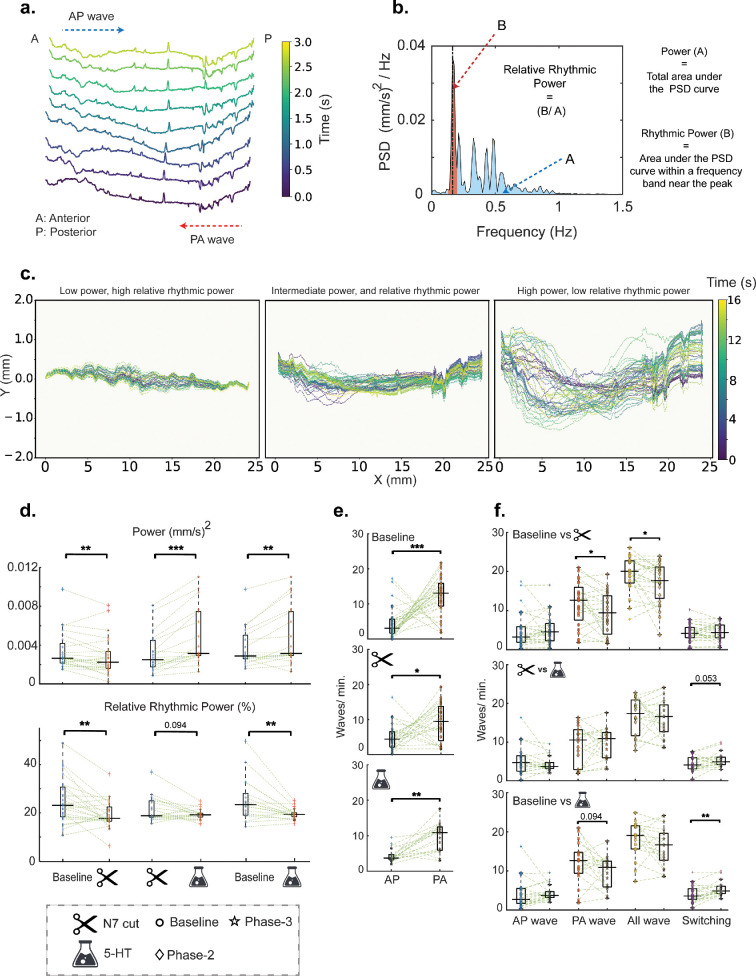
DV motility and lateral waves after denervation, and serotonergic modulation. (a) Depicting the temporal evolution of gut motility patterns over a 3-s window, with colour intensity representing the progression of time. (b) Defining power and relative rhythmic power from a demonstrative PSD curve. (c) Demonstrating representative examples of motility patterns that correspond to different degrees of power and rhythmic power. (d) Boxplots showing variation in total power and relative rhythmic power with different treatments. To test for statistical significance, movies with similar treatments were pooled. For 5-HT experiments, the number of repeats was *n* = 6 for each condition 1, 10 and 100  μm). Comparisons between baseline and N7 cut included all N7 cut movies (*n* = 24). Comparisons between N7 cut and 5-HT, as well as Baseline and 5-HT, were conducted using the pooled 5-HT dataset (*n* = 18). Each dot represents the corresponding power or relative rhythmic power values for the chosen 15-min interval of a unique movie. Relative rhythmic power = area under the PSD at peak frequency ± 0.04 Hz/total power. (e) Number of AP versus PA waves during the three phases of the experiments (baseline, N7 cut and 5-HT), pooling all movies with similar treatments. Sample sizes: Baseline (*n* = 30), N7 cut (*n* = 24) and 5-HT (*n* = 18). (f) Boxplots showing the variation in the number of AP waves, PA waves, total waves and directional switching events across different treatments. For baseline versus N7 cut comparisons, all N7 cut movies were used (*n* = 24). For both baseline versus 5-HT and N7 cut versus 5-HT comparisons, pooled 5-HT experiment movies were used (*n* = 18). *p*-values were obtained using Friedman and Wilcoxon’s signed rank test criteria. Total number of waves = no. of AP waves + no. of PA waves + no. of Mixed waves. Boxes indicate the interpolated 25th−75th percentiles of the sample data, and the horizontal solid black line indicates the median value.

Since circular contractions are limited, tracking the central axis provides a reasonable way to capture gut motility (figure 4a). However, total power (figure 4b) does not fully capture the intricate ST patterns of movement observed. We found that different regions of the hindgut can exhibit varying frequencies of movement, leading to standing wave patterns due to the fixed endpoints. A regular, periodic motion is characterized by a sharp peak in the power spectral density (PSD) at a specific frequency (rhythmic component) with minimal contributions from other frequencies (non-rhythmic component). Conversely, a PSD with multiple broad peaks indicates irregular, arrhythmic motion. With this reasoning, we used the dimensionless metric ‘relative rhythmic power’ (interchangeably used with ‘rhythmic power’), to quantify the temporal coordination of motion (figure 4b). Figure 4c shows representative examples of central axis motion over time, corresponding to varying levels of total power and rhythmic power. Next, we pooled movies with similar treatments and performed pairwise statistical tests to identify significant differences in motility parameters.

For 5-HT experiments, we found a significant difference in overall power between all three phases (figure 4d). As shown previously in figure 3, we noticed that power decreased upon denervation (*p* = 0.0061) and then increased again upon 5-HT application (*p* = 0.00023) which was higher even than the baseline (*p* = 0.0043). Similarly, we observed the fraction of rhythmic power decreasing upon denervation in a statistically significant manner (*p* = 0.007*)*. However, unlike overall power, it stayed significantly lower (*p* = 0.005) than baseline levels even after the application of 5-HT. Electronic supplementary material, figure S3 shows that there is no significant overall trend in the dose-dependent effects of 5-HT on rhythmic power across the treatments.

Relative rhythmic power is a characteristic of coordinated movement in the frequency or time domain. In figure 2, we observed spatial coordination of the gut, resulting in wave-like motility patterns. To capture this coherence and further augment our frequency-space analysis, we applied our wave-identification workflow to all motility plots. With the instantaneous DV speed and spatial wave-span thresholds, we extracted lateral gut waves travelling in both directions. We also studied the directional switching events for such waves and the potential effects of external perturbations on them. For the 5-HT experiments, we observed a strong preference for PA over AP movement irrespective of the treatment (figure 4e); *p* = 0.00042 for baseline, *p* = 0.019 upon denervation and *p* = 0.0012 after the application of 5-HT. The total number of waves (*p* = 0.022) and the number of PA waves (*p* = 0.04) both decreased upon N7 cut (figure 4f) but did not rise back up by a significant amount upon application of 5-HT. We also noted, upon the application of 5-HT, the number of directional switching of lateral waves (AP → PA and PA → AP, combined) increased significantly (*p* = 0.01) with respect to the baseline (figure 4f) and slightly (but statistically non-significant, *p* = 0.053) compared to the N7 cut phase. N7 cut did not result in any significant change in switching wave directions compared to the baseline. The number of AP waves was not affected by any treatment.

### 3.4. TTX blocks electrophysiological activity but does not fully suppress mechanical activity

To investigate the relative contributions of neural and myogenic components to hindgut motility, we performed experiments with TTX, a potent blocker of voltage-gated Na^+^ channels.

**Figure 5.**
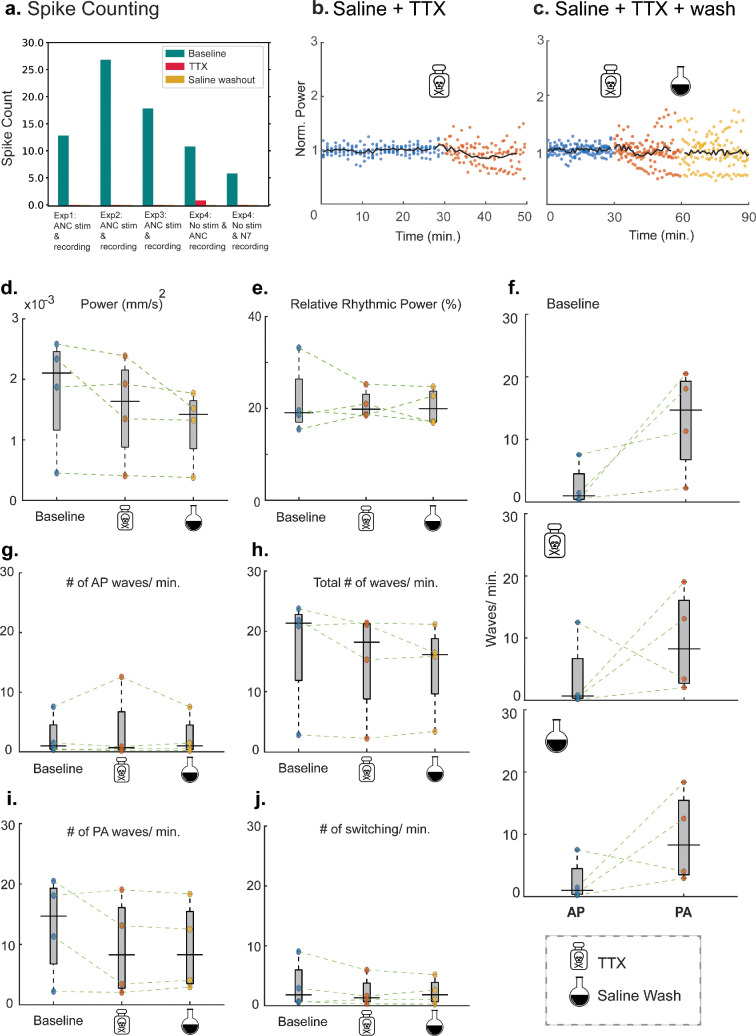
Effects of TTX modulation on hindgut motility metrics. (a) Number of spikes recorded during 15- min time windows 15–30, 45−60 and 105−120 min). Number of repeats for TTX + saline wash experiments: *n* = 4. The recordings were performed either in the ANC (abdominal nerve cord) or N7. One experiment was without external stimulation. (b,c) Normalized power versus time for all TTX experimental runs. *n* = 6 for saline + TTX and *n* = 5 for saline + TTX + wash. TTX and saline profusion were administered at 30 and 60 min from the start, respectively. (d,e) Boxplots showing variation in total power and relative rhythmic power with TTX experiments. (f) No. of AP versus PA waves during three different phases of the TTX experiments (baseline, TTX, saline wash). (g,h) Boxplots showing variation in the no. of AP, and total waves with TTX treatments. (i,j) Boxplots depicting the number of PA waves, and the number of directional switching events across TTX experiments. Total number of waves = no. of AP waves + no. of PA waves + no. of mixed waves. Boxes indicate the interpolated 25th−75th percentiles of the sample data, and the solid black line indicates the median value.

Thus, changes in gut motility after TTX treatment would indicate involvement of neurogenic mechanisms rather than intrinsic myogenic control. As expected, TTX treatment significantly reduced neuronal activity, as evidenced by a dramatic decrease in spike counts which could not be recovered after saline wash (figure 5a). Despite blocking neural activity, we observed a persistent level of coherent gut motility after TTX and saline wash, as indicated by non-zero power and relative rhythmic power values (figure 5b–e). Furthermore, analysis of lateral wave propagation revealed that the dominant directionality (posterior to anterior) was maintained even after TTX treatment (figure 5f). The number of PA waves and total number of waves decreased slightly (figure 5i,h). The application of TTX did not induce a substantial change in the frequency of directional switching events (figure 5j). For the control condition, we observed sustained neurophysiological activity throughout 90 min showing the viability of the preparation (electronic supplementary material, figure S4).

## 4. Discussion

The crayfish *ex vivo* hindgut-nerve cord preparation and analysis framework we developed offers a useful model to advance our understanding of CNS regulation and interrelated neurochemical modulation of GI motility. The hindgut displayed steady power dynamics during 90 min of saline perfusion, allowing us to investigate transient changes after denervation and subsequent application of 5-HT. Moreover, our analysis framework was non-invasive, solely using brightfield images to study lateral movements of the hindgut. Using optical flow analysis allowed us to detect and quantify movement at pixel (equivalent to μm scale) resolution along a significant length of the hindgut (24.2 ± 0.93 mm, or approx. 50% total hindgut). This method, coupled with Fourier transformation further provided us with the tools to distinguish between different regimes of lateral hindgut motility, such as rhythmic, regular movement and more uncoordinated, irregular motion. Using multiple parameters to characterize gut motility, such as frequency, total power, rhythmic power and wave direction, we were able to discriminate among the different modulating systems of hindgut motility, including the motion driven by intrinsic putative myogenic pacemakers of the hindgut [18], the CNS, and associated serotonergic effects.

While previous work in animal models has focussed extensively on wave behaviours in short intestinal segments innervated by the ENS, few methods exist for directly tracking whole gut motility in concert with CNS regulation and serotonergic modulation while providing continuous spatial localization of these movements [35,40–42]. Our model provides a simpler anatomical platform than traditional vertebrate models for analysing this interplay continuously across organ systems [43]. Moreover, unlike existing techniques such as force transducers or motion analysis of short intestinal segments, this platform aggregates data across multiple scales, allowing detection of local effects as well as visualizing global behaviours. In the future, concurrent electrophysiology of N7 neurons could be combined with neuropharmacology and optical flow video analysis to expand the modalities of our multiscale analysis framework.

Our study’s bath application of 5-HT after CNS denervation most closely mimicked the physiological effects of *in vivo* neuro-hormonal 5-HT release that reaches the crayfish hindgut via the dorsal abdominal artery that runs along it [19,44]. While the concentrations of 5-HT (1–100 μM) and rate of application (5 ml min^−1^) used for this study were chosen based on their reported excitatory effects on crayfish lateral giant neurons [45] and increased power of middle and caudal hindgut pulsatile contractions [34], the physiological concentration of 5-HT in the haemolymph of live crayfish may be lower [46]. Tonic levels of 5-HT have been measured in the haemolymph of similar organisms such as American lobsters [47] and green crabs [48] at 1 and 0.5 nM, respectively. However, systemic measurements may differ from local release concentrations and other possible sources of 5-HT may exist in the hindgut. Interestingly, crayfish hindgut neurons have been shown to lack the ability to synthesize 5-HT and have been posited to borrow 5-HT through uptake from the extracellular medium [19]. Moreover, other sources of 5-HT, like the mammalian enterochromaffin cells in the gut epithelium that produce more than 90% of 5-HT in the body [24] may be present in the crayfish hindgut, but this remains to be further studied.

Notably, changes in hindgut power after 5-HT application showed dose-dependent effects. Hindgut motility power increased linearly with 5-HT concentration and returned close to CNS-regulated baseline levels depending upon the 5-HT concentration. Particularly, hindgut motility power was the parameter notably potentiated by application of serotonin at higher doses while decreasing after prolonged application (figure 3), possibly [49,50] related to differences in binding affinity of different 5-HT receptors. However, bath application of 5-HT may not reflect the fine-tuned actions of serotonergic hindgut neurons that originate in the abdominal ganglion [34]. Although these neurons target spatially differentiated 5-HT1α and 5-HT2β receptors along the hindgut, it remains unclear whether they participated in CNS-derived regulation of gut motility because we did not measure synaptic 5-HT release. Furthermore, the longitudinal and circular muscles producing lateral waves might also respond differently to 5-HT as reported for neuropeptides in crayfish [22]. Thus, failure to recover ST coordination after ‘neurohormonal’ bath application of 5-HT points to the absence of coordination in sequential activation of these 5-HT receptors.

Our results suggest an active role of hormonal 5-HT (via bath application) in modulating hindgut motility power in a linearly dose-dependent manner, while the CNS ensures overarching coordination of movement in space and time. This is supported by our findings that both N7 denervation and 5-HT application independently caused significant changes in temporal regularity (rhythmic power) and in the amount and power of lateral waves. However, to understand whether CNS-derived modulation is related to synaptic 5-HT release from hindgut neurons or due to other neurotransmitters or modulatory mechanisms further pharmacology experiments are needed. Our TTX experiments suggest a more prominent and intricate involvement of myogenic components in regulating gut motility compared to the neurogenic components. Despite its neurotoxic potency, there was no significant difference observed in the overall extent as well as ST coordination (electronic supplementary material, figure S2) of gut motility before and after the addition of TTX. These findings suggest that while neuronal input plays a significant role in regulating hindgut motility, intrinsic myogenic mechanisms can generate and sustain rhythmic contractions, even in the absence of neural activity. Moreover, intrinsic mechanisms such as myogenic pacemakers likely control the parameters that did not show changes after denervation, namely wave directional preference and frequency, the latter staying within the previously reported [19,34] range of 0.2−0.4 Hz (see electronic supplementary material, figure S2).

These intrinsically controlled parameters are probably determined by the animals’ internal states, such as feeding status (all animals were food deprived 3−4 days before experiments). In most experiments, we observed a directional preference for hindgut lateral waves traveling from PA rather than the reverse direction. This is supported by previous work in crayfish that reported mostly PA or ‘antiperistaltic’ waves in the denervated, empty hindgut, while AP or peristaltic contractions were only observed by introducing a small wax bead into the gut lumen [30] or in lobster, by supplying central input from the terminal abdominal ganglion to initiate the defecation reflex [14]. Like antegrade peristalsis which has also been reported in intestinal segments of vertebrates, crayfish hindgut antiperistalsis may play similar roles in content mixing, osmoregulation, and gut microbiota regulation [51–55]. Further studies of crayfish hindgut motility may improve our understanding of bidirectional wave propagation and its interplay with CNS regulation.

Finally, our study provides an especially simple anatomical model to study the bidirectional interplay between the CNS and intestinal motility [51,56]. In recent years, there has been a high level of interest in the general health effects of the gut microbiome in modulating the brain [57,58]. Particularly, the interplay of the gut microbiome–brain axis and intestinal motility is an exciting avenue for future research in the crayfish model [59–61].

## Data Availability

Data is available online [62]. Supplementary material is available online [63].
